# Single-Center Retrospective Analysis of Risk Factors for Hydrocephalus After Lateral Ventricular Tumor Resection

**DOI:** 10.3389/fsurg.2022.886472

**Published:** 2022-06-16

**Authors:** Chengda Zhang, Lingli Ge, Zhengwei Li, Tingbao Zhang, Jincao Chen

**Affiliations:** ^1^ Department of Neurosurgery, Zhongnan Hospital of Wuhan University, Wuhan, China; ^2^ Department of Neurosurgery, The Affiliated Hospital of Hubei University of Medicine, The First People’s Hospital of Xiangyang, Xiangyang, China; ^3^ Department of Paediatrics, Xiangyang Central Hospital, Affiliated Hospital of Hubei University of Arts and Science, The Central Hospital of Xiangyang, Xiangyang, China

**Keywords:** lateral ventricular tumor, CSF drainage, hydrocefalus, EVD, VP shunt

## Abstract

**Objective:**

There is no general consensus on the placement of preoperative and intraoperative external ventricular drainage (EVD) in patients with lateral ventricular tumors (LVTs). The aim of this study was to identify the predictors of postoperative acute and persistent hydrocephalus need for postoperative cerebrospinal fluid (CSF) drainage and guide the management of postoperative EVD in patients with LVTs.

**Methods:**

We performed a single-institution, retrospective analysis of patients who underwent resection of LVTs in our Department between January 2011 and March 2021. Patients were divided between one group that required CSF drainage and another group without the need for CSF drainage. We analyzed the two groups by univariate and multivariate analyses to identify the predictors of the requirement for postoperative CSF drainage due to symptomatic intracranial hypertension caused by hydrocephalus.

**Results:**

A total of 97 patients met the inclusion criteria, of which 31 patients received preoperative or intraoperative EVD. Ten patients without prophylactic EVD received postoperative EVD for postoperative acute hydrocephalus. Eleven patients received postoperative ventriculoperitoneal(VP) shunt subsequently. Logistic regression analysis showed that tumor invasion of the anterior ventricle (OR = 7.66), transependymal edema (OR = 8.76), and a large volume of postoperative intraventricular hemorrhage (IVH) (OR = 6.51) were independent risk factors for postoperative acute hydrocephalus. Perilesional edema (OR = 33.95) was an independent risk factor for postoperative VP shunt due to persistent hydrocephalus.

**Conclusion:**

Postoperative hydrocephalus is a common complication in patients with LVTs. These findings might help to determine whether to conduct earlier interventions.

## Introduction

Ventricular tumors refer to lesions originating in related ventricular structures or secondary ventricular neoplasms originating in periventricular tissues and most of the neoplasms (more than 2/3) invading the ventricle (1). Resection is the main treatment measure for ventricular tumors (2). Tumor resection can restore the cerebrospinal fluid (CSF) circulation pathway, with some lateral ventricular tumor (LVT) patients showing relief of intracranial hypertension symptoms after resection. However, some patients can develop secondary symptomatic intracranial pressure (ICP) elevation after resection, with a requirement for CSF drainage (3). Placement of external ventricular drainage (EVD) is necessary for patients with acute hydrocephalus. Although an intraoperative lateral ventricular drainage tube is convenient to place along the intracranial surgical pathway after tumor resection, most postoperative patients would not develop clinical symptoms with intracranial hypertension and need CSF drainage. We can also not ignore the complications of EVD placement such as CSF leak, infection, and so on. Now, there is no consensus on the indication for the placement of preoperative and intraoperative EVD. Studies without the use of regression analysis suggest that hydrocephalus after LVT resection may be related to the surgical approach, tumor location, degree of resection, and displacement of hemostatic material (4–6). Thus, the aim of this study was to identify the predictors associated with intracranial hypertension in need of CSF drainage after LVT resection using regression analysis and guide the management of EVD placement.

## Materials and Methods

### Study Population and Data Collection

This retrospective descriptive cohort study investigated the incidence and predictors of postoperative acute and persistent hydrocephalus in a consecutive group of patients who underwent surgery for LVT between January 2011 and February 2021 in the Department of Neurosurgery at Zhongnan Hospital of Wuhan University. Patients diagnosed with LVTs by outpatient computerized tomography (CT) or magnetic resonance imaging (MRI) were admitted to the Neurosurgery Department, Zhongnan Hospital of Wuhan University. The exclusion criteria were (1) patients who underwent biopsy or did not receive tumor resection; (2) patients with multiple intracranial tumors; (3) patients presenting for biopsy; (4) patients who received a ventriculoperitoneal (VP) shunt or endoscopic third ventriculostomy (ETV) before tumor resection; and (5) the space-occupying lesion confirmed not to be a tumor by histological diagnosis. Clinical information was recorded for all enrolled with a follow-up duration ranging from 90 days to 6 years.

Clinical data and imaging materials collected were obtained from the hospital’s electronic database. We recorded information on sex, age, tumor location, tumor size, tumor histology, the presence of perioperative hydrocephalus, resection degree, whether a hemostatic agent such as gel foam was placed in the lateral ventricle during surgery, the volume of postoperative intraventricular hemorrhage (IVH), perioperative placement of EVD, VP shunt, and ETV, and the volume of CSF drainage every day.

The diagnostic criteria for preoperative hydrocephalus were imaging results indicating the Evans index (the maximum width between the frontal horns divided by the maximal width of the inner table) ≥30%, with signs of intracranial hypertension such as headache, nausea, visual deterioration, etc. (7).

The criteria for preoperative EVD placement were radiographic diagnosis of hydrocephalus with a decreased level of consciousness at admission due to severe cranial hypertension. If the patient's decrease of consciousness is caused by thalamus invasion or cerebral herniation, we usually choose to remove the lesion directly. Before resection, CSF was drained via the EVD and the ICP was controlled in the normal range to stabilize these patients. For patients with mild clinical symptoms of intracranial hypertension, we would complete preoperative preparation and perform surgery as soon as possible to relieve intracranial hypertension. Whether to place an intraoperative EVD was decided by surgeons during surgery. We tend to place an intraoperative EVD in patients with tumors that have rich blood supply or no clearly distinct boundaries. A patient who received a preoperative EVD would no more receive an intraoperative EVD.

All postoperative patients would stay in the Neurosurgical Care Unit (NCU) for at least 24 h. After resection, the EVD was opened most of the time and the drain height was set over 20 cm above the level of the external auditory meatus ordinarily. The amount of CSF drained every day was recorded. Normally, there would be a little volume of drainage. The drainage tube was clamped intermittently to calculate the pressure equalization ratio (8). The ICP was continuously monitored. Once the patient developed clinical symptoms of intracranial hypertension such as vomiting, nausea, or impaired consciousness, with the ICP increased above 20 mm Hg with the EVD opened or intermittently closed and without physiological influence such as cough, the drain height was decreased and CSF was drained via the EVD to smoothly decrease the ICP to the normal range.

The criteria for a postoperative EVD placement were acute hydrocephalus with symptomatic intracranial hypertension developed such as vomiting, nausea, or impaired consciousness, and Evans ratio ≥30% (9). All patients with postoperative acute hydrocephalus received an EVD placement in our department.

The time for drainage removal was <14 days. The criteria for drainage removal are as follows: (1) the patient was in a stable condition, with increasing drainage height over a few days followed by closing the drainage tube for at least 12 h without intracranial hypertension symptoms, postoperative CSF leakage, and subcutaneous effusion; and (2) the CT scan was negative for 24 h before drainage removal.

The criteria for a postresection VP shunt were failed EVD weaning, symptomatic chronic hydrocephalus, stubborn CSF fistula with intracranial hypertension, or an isolated ventricle requiring persistent CSF drainage.

**Figure 1:**
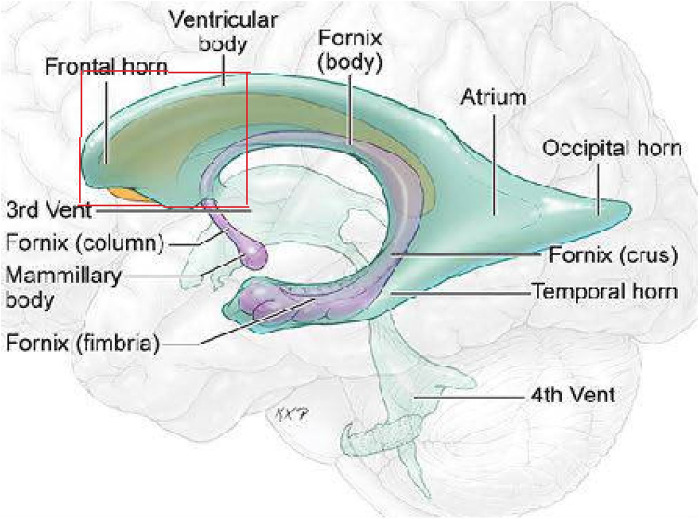
Red box showing the anterior part of the lateral ventricle.

**Figure 2:**
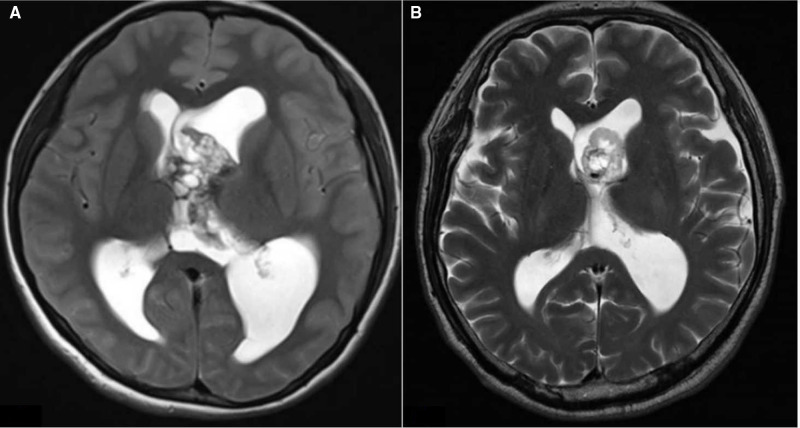
(**A, B**) Axial T2 images showing the anterior invasion of lateral ventricular tumors.

On the basis of preoperative MRI, we classified LVTs as either anterior invasion tumors (i.e., tumors invading the anterior part of the lateral ventricle (Figure 1) or tumors without anterior invasion (i.e., tumors not invading the anterior part of the lateral ventricle). MRI images show a typical case invading the anterior part of the lateral ventricle (Figure 2).

Tumor volume was measured by MRI using the formula of ABC/2 (10). At our center, during the resection of the tumor of a big size, we routinely use the technology of ultrasonic aspiration or/and assisted endoscopic resection to increase the surgical success rate and decrease the operation time. It means that large-sized tumors can indirectly represent a more difficult resection.

Perilesional and transependymal edema was recorded from the preoperative MRI scan. Transependymal edema was defined as periventricular hyperdense signals around the posterior and particularly the anterior horns in T2-weighted images with a thickness of ≥3 mm (11). Perilesional and thin periventricular hyperintensity was not considered to be transependymal edema. As a brain tumor-associated perilesional edema is uniquely responsive to corticosteroids, corticosteroids are used only when the effect is poor after measures including elevation of the head of the bed, fluid restriction, mannitol use, and diuretics are taken.

Total resection was defined as no residual tumor tissue being seen by surgeons after resection and no reactive enhancement within 72 h after surgery on MRI (12).

Large volume of postoperative IVH was defined according to the IVH score as at least more than half of one lateral ventricle filled with blood, with or without the third or fourth ventricle filled with blood partly or completely (13).

Patients who needed CSF drainage due to intracranial hypertension in combination with relevant clinical symptoms were grouped in the CSF drainage group. Patients who received a prophylactic EVD but did not need to drain CSF were grouped into the non-CSF drainage group.

### Study Design

All patients after resection were grouped into either the CSF drainage group or the non-CSF drainage group. The primary and secondary endpoints of the study were to determine the predictors of postoperative acute hydrocephalus and persistent hydrocephalus, respectively. We used median values to analyze age and tumor size. We dichotomized the clinical and radiological parameters. EVD was placed in all patients with postoperative acute hydrocephalus, and all enrolled patients who developed postoperative persistent hydrocephalus received a VP shunt. All cases were analyzed together to determine the predictors of postoperative acute hydrocephalus. We analyze the rest of cases after eliminating the cases of isolated hydrocephalus to determine the predictors of postoperative persistent hydrocephalus.

This study was approved by the Ethics Committee of Zhongnan Hospital of Wuhan University. The study did not involve patients’ personal or private information, and patient consent was therefore not required.

### Statistical Analysis

Data were analyzed using statistical software (IBM SPSS Statistics v22 and v24). Student’s *t*-test was used for comparisons between the two groups. Binary parameters were analyzed with a chi-square test. Multivariate logistic regression analysis was performed to find independent predictors of EVD placement. Odds ratios (ORs) and 95% confidence intervals were calculated to assess the impacts of the variables. *P* < 0.05 was considered to be statistically significant.

## Results

### Patient Characteristics

**Figure 3:**
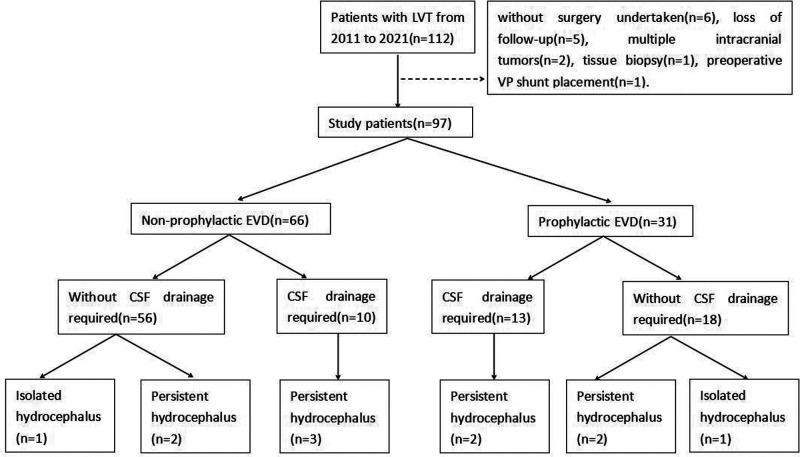
Flow chart for the study population.

A total of 97 patients were finally enrolled in this study. Fifteen patients were excluded, including six patients without surgery, five patients with the loss of follow-up, two patients with multiple intracranial tumors, one patient with a biopsy, and one patient with a preoperative VP shunt (Figure 3).

The average age of the enrolled patients was 42.2 years (range, 6–79 years), with 47 male patients (48.5%) and 50 female patients (51.5%). The result of tumor histology showed 32 patients with meningioma, 21 patients with central neurocytoma, 18 patients with glioma, 11 patients with ependymoma, 5 patients with choroid plexus tumor, 3 patients with hemangioma, 2 patients with germinoma, and 5 patients with other pathological results. Eleven patients received a VP shunt after resection latterly. The tumor histological characters and location data of the patients are shown in Table 1.

**Table 1:** Location and histological characters of LVTs with postoperative CSF drainage.

	Number	Postoperative CSF drainage required by EVD		Postoperative CSF drainage required by a VP shunt	
		Anterior invasion	No anterior invasion	Anterior invasion	No anterior invasion
Meningoma	32(33.0%)	0	3(42.9%)	0	1(16.7%)
Central neurocytoma	21(21.6%)	7(43.8%)	2(28.6%)	0	3(50%)
Glioma	18(18.6%)	9(56.2%)	1(14.3%)	4(80%)	2(33.3%)
Ependymoma	11(11.3%)	0	0	0	0
Choroid plexus tumor	5(5.2%)	0	1(14.3%)	1(20%)	0
Cavernous angioma	3(3.1%)	0	0	0	0
Germinoma	2(2.1%)	0	0	0	0
Other	5(5.2%)	0	0	0	0
Total	97	16	7	5	6

*CSF, cerebrospinal fluid; EVD, external ventricular drainage; VP, ventriculoperitonea.*

### Predictors of Postoperative Acute Hydrocephalus Required CSF Drainage by EVD

Overall, 31 patients received a prophylactic EVD placement, 13 of which needed CSF drainage after tumor resection. Ten patients without prophylactic EVD developed acute hydrocephalus with symptomatic intracranial hypertension and received postoperative EVD. In total, 23 patients needed CSF drainage after tumor resection (range, 12–72 years). This accounted for 41.9% (13/31) of the patients with prophylactic EVD and 15.2% (10/66) of the patients without prophylactic EVD.

Univariate analysis showed that anterior invasion (*P* < 0.001), preoperative hydrocephalus (*P* = 0.008), intraventricular hemostatic agent (*P* < 0.001), large volume of postoperative IVH (*P* = 0.02), transependymal edema (*P* < 0.001), perilesional edema (*P* = 0.001), and glioma (*P* = 0.006) were predictive factors of the need for postoperative acute hydrocephalus requiring CSF drainage by EVD (Table 2).

**Table 2:** Summary of the factors with postoperative acute hydrocephalus required CSF drainage by EVD.

	Postoperative acute hydrocephalus		*P* value
	Yes	No	
Sex			0.95
Male	11(11.3%)	36(37.1%)	
Female	12(12.4%)	38(39.2%)	
Age (≤18 years)			0.34
Yes	3(%)	5(%)	
No	20(%)	69(%)	
Tumor size (cm3)	31.9 ± 31.5	20.7 ± 16.7	0.055
Anterior invasion			<0.001
Yes	16(16.5%)	14(14.4%)	
No	7(7.2%)	60(61.9%)	
Large volume of postoperative IVH			0.02
Yes	9(9.3%)	12(12.4%)	
No	14(14.4%)	62(63.9%)	
Preoperative hydrocephalus			0.008
Yes	16(16.5%)	28(28.9%)	
No	7(7.2%)	46(47.4%)	
Subtotal resection			0.18
Yes	5(5.2%)	8 (8.2%)	
No	18(18.6%)	66 (68%)	
Perilesional edema			0.001
Yes	20(20.6%)	18(18.6%)	
No	3(3.1%)	56(55.7%)	
Transependymal edema			<0.001
Yes	15(15.5%)	5(5.2%)	
No	8(8.2%)	69(71.1%)	
Intraventricular hemostatic agent			<0.001
Yes	13(13.4%)	15(15.5%)	
No	10(10.3%)	59(60.8%)	
Histology			
Meningoma	3(3.1%)	29(29.9%)	0.08
Central neurocytoma	9(9.3%)	12(12.4%)	0.07
Glioma	10(10.3%)	8(8.2%)	0.006
Ependymoma	0(0)	11(11.3%)	0.07
Choroid plexus tumor	1(1%)	4(4.1%)	0.85
Cavernous angioma	0(0)	3(3.1%)	0.34
Germinoma	0(0)	2(2.1%)	0.43
Other	0(0)	5(5.2%)	0.22

*CSF, cerebrospinal fluid; EVD, external ventricular drainage; IVH, intraventricular hemorrhage*.

Multivariate analysis identified anterior invasion (OR = 7.42), large volume of postoperative IVH (OR = 6.46), and transependymal edema (OR = 8.7) as independent risk factors of postoperative acute hydrocephalus requiring CSF drainage by EVD (Table 3).

**Table 3:** Multivariate analysis of the association between factors and postoperative acute hydrocephalus required CSF drainage by EVD.

	Multivariate analysis	
	*P* value	OR(95%CI)
Anterior invasion	0.016	7.42(1.45–38.01)
Large volume of postoperative IVH	0.022	6.46(1.3–32.01)
Transependymal edema	0.045	8.7(1.05–72.36)
Perilesional edema	0.056	–
Preoperative hydrocephalus	0.98	–
Intraventricular hemostatic agent	0.63	–
Glioma	0.006	–

*Nagelkerke R2 = 0.56.*

*CSF, cerebrospinal fluid; EVD, external ventricular drainage; IVH, intraventricular hemorrhage.*

In this study, the histological characters of LVT were not considered to be a risk factor for postoperative EVD placement.

### Predictors of Persistent Hydrocephalus Required CSF Drainage by a VP Shunt

**Figure 4:**
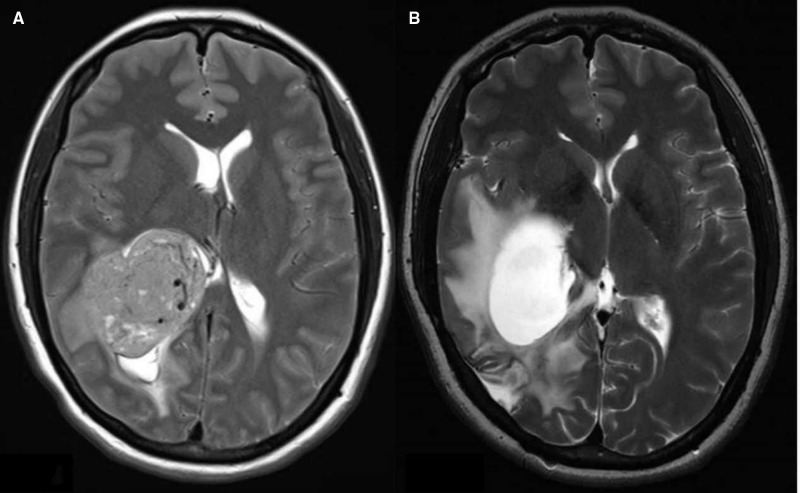
(**A**). Preresection axial T2 image showing the right lateral ventricular tumor, (**B**). Postresection axial T2 image showing the isolated hydrocephalus.

Eleven (11.3%) of all patients received a VP shunt postoperatively, including two (2.1%) patients with a prophylactic EVD. The mean implantation time was 62.4 days (range, 14–191 days). There were five patients with obstructive hydrocephalus, four patients with communicative hydrocephalus, and two patients with isolated hydrocephalus. Both patients with postresection isolated hydrocephalus had tumors at the occipital angle of the lateral ventricle. It was difficult to achieve complete resection during surgery for the first patient with the tumor histology of glioblastoma. The postoperative MRI revealed that the obstruction was not optimally removed because of the occupying effect and adhesion of the residual tumor tissue. Then, a VP shunt was inserted due to unrelieved symptomatic intracranial hypertension. The second patient showed a meningioma with complete intraoperative resection. However, postoperative MRI showed obstruction caused by cerebral tissue adhesion accompanied by intracranial hypertension (Figure 4). The two patients with isolated hydrocephalus were excluded from the data analysis.

Univariate analysis showed that tumor size (*P* = 0.003), preoperative hydrocephalus (*P* = 0.01), subtotal resection (*P* = 0.003), transependymal edema (*P* = 0.017), glioma (*P* = 0.022), and perilesional edema (*P* = 0.015) were predictive factors for a postresection VP shunt (Table 4).

**Table 4:** Summary of the factors with a postoperative VP shunt.

	Posotoperative VP shunt		*P* value
	Yes	No	
Sex			0.39
Male	4(4.1%)	43(44.3%)	
Female	5(7.2%)	43(44.3%)	
Age (≤18 years)			0.65
Yes	1	6	
No	8	80	
Tumor size (cm3)	54.9 ± 53.7	19.5 ± 12.8	0.003
Anterior invasion			0.33
Yes	2(2.1%)	28(29.5%)	
No	7(7.4%)	58(59.8%)	
Large volume of postoperative IVH			0.63
Yes	3(3.2%)	18(18.9%)	
No	6(6.3%)	68(71.6%)	
Preoperative hydrocephalus			0.01
Yes	8(8.4%)	35(36.9%)	
No	1(1.1%)	51(53.7%)	
Subtotal resection			0.001
Yes	5(5.3%)	8(8.4%)	
No	4(4.2%)	78(82.1%)	
Postoperative acute hydrocephalus			0.3
Yes	5(5.3%)	18(18.9%)	
No	4(4.2%)	68(71.6%)	
Perilesional edema			0.015
Yes	7(7.4%)	30(31.6%)	
No	2(2.1%)	56(58.9%)	
Transependymal edema			0.017
Yes	6(6.3%)	24(25.3%)	
No	3(3.2%)	62(65.3%)	
Intraventricular hemostatic agent			0.2
Yes	5(5.3%)	23(24.2%)	
No	4(4.2%)	63(66.3%)	
Histology			
Meningoma	0(0)	31(32.6%)	0.075
Central neurocytoma	3(3.2%)	18(18.9%)	0.51
Glioma	5(5.3%)	12(12.6%)	0.022
Ependymoma	0(0)	11(11.6%)	0.29
Choroid plexus tumor	1(1.1%)	4(4.2%)	0.44
Cavernous angioma	0(0)	3(3.2%)	0.58
Germinoma	0(0)	2(2.1%)	0.65
Other	0(0)	5(5.3%)	0.47

*IVH, intraventricular hemorrhage; VP, ventriculoperitonea.*

Multivariate analysis identified perilesional edema (OR = 33.95) as an independent negative predictor for a postresection VP shunt (Table 5).

**Table 5:** Multivariate analysis of the association between a postoperative VP shunt and factors.

	Multivariate analysis	
	*P* value	OR(95%CI)
Tumor size (cm^3^)	0.58	–
Subtotal resection	0.06	–
Preoperative hydrocephalus	0.74	–
Transependymal edema	0.11	–
Glioma	0.022	–
Perilesional edema	0.04	33.95(1.17–998.78)

*Nagelkerke R^2^ = 0.566.*

*VP, ventriculoperitonea.*

## Discussion

Patients in whom the ventricles were entered during surgery had significantly higher rates of postoperative hydrocephalus, EVD placement, and VP shunt placement (14). Especially for patients with LVTs, there is no current consensus on the optimal placement of prophylactic EVD for these patients. Placement of a prophylactic EVD is used to drain residual hematoma clots and bloody and protein-rich CSF, as well as to monitor and regulate ICP. Drainage placement can also prevent intracranial hypertension caused by acute hydrocephalus. Intraoperative EVD placement is convenient for surgeons to undertake as the tube can be placed through the surgical channel after tumor resection. By contrast, for patients without prophylactic EVD who develop postoperative acute hydrocephalus, the ventricular puncture site for EVD placement is selected in the emergency unit, which increases the risks of brain injury and other morbidities. Nevertheless, there are also some risks of intraoperative EVD placement, including intracranial infection, increased risk of CSF leakage, and potential risk of excessive drainage. The aim of the present study was to identify patients at high risk of acute hydrocephalus after LVT surgery for the guidance of prophylactic EVD placement and to analyze the risk factors for postresection VP shunt placement.

Our center recommends an EVD insertion first rather than an early or direct VP shunt placement when postoperative acute hydrocephalus occurs. In this study, 5/23(21.7%) patients with postoperative acute hydrocephalus then received a VP shunt due to persistent hydrocephalus. Persistent hydrocephalus may be caused by the persistent obstruction of the arachnoid granulations by blood-breaking products and their inflammatory reactions (15). Postoperative acute hydrocephalus can resolve in most cases and would not develop onto persistent hydrocephalus with the insertion of EVD.

To the best of our knowledge, this regression analysis is the first to identify the characteristics of tumor location, perilesional edema, intraventricular hemostatic agent, and other predictors for hydrocephalus after LVT resection. We found that tumor invasion of the anterior part of the lateral ventricle was an independent risk factor for postoperative CSF drainage caused by acute symptomatic hydrocephalus. Deling et al. reported that hydrocephalus tends to develop after an LVT resection in which the tumor basement is located at the lateral ventricular wall, dorsal thalamus, choroid plexus, or third ventricle (near the foramen of Monro) (4), but statistical confirmation was not performed in their study. Anatomically, the posterior internal choroid artery expands radially through the foramen of Monro and is the main blood supply vessel of the anterior part of the ventricle (16). During resection of tumors located at or invading the anterior part of the lateral ventricle, damage to these branching vessels may increase brain tissue swelling around the midbrain aqueduct after surgery, thereby narrowing the CSF circulation pathway. For tumors invading the anterior ventricle wall or the aqueduct of the lateral ventricle, postoperative tissue adhesion may cause obstruction (17). Ktari et al. reported that postoperative obstructive hydrocephalus can be caused by the displacement of intraventricular hemostatic materials and the inflammatory reaction associated with Gelfoam residue, with a surrounding marked giant cell reaction with underlying fibrosis, thrombosis of small superficial vessels, and reactive microglials (6).

In the present study, large volume of postoperative IVH was also an independent risk factor for postoperative CSF drainage due to acute symptomatic hydrocephalus. Postoperative hemorrhage is a serious complication after LVT resection, which typically manifests as intraventricular hemorrhage, while approximately 50% of patients with IVH will develop hydrocephalus (18, 19). Importantly, blood can stimulate the production of CSF (17), while the mass effect of the hematoma can obstruct the CSF circulation pathway and cause symptoms of intracranial hypertension, which requires CSF drainage (20, 21).

Intracranial tumors with perilesional edema are more difficult to remove and mostly malignant. These tumors are more likely to develop severe focal edema after surgery. Perilesional edema in LVTs appear as paraventricular edema. After tumor resection, the CSF pathway can be more negatively affected in LVTs with perilesional edema.

It have been proposed that the major CSF pathway consists of the circulation of CSF from the lateral ventricles to the third ventricle via the foramen of Monro, and the minor pathway of CSF leads through the ventricular ependymal layer, interstitial and perivascular space, and perineural lymphatic channels (22). Transependymal edema may be caused by increased intraventricular pressure and impaired function of the minor CSF pathway (11). Therefore, for patients with preoperative communicative hydrocephalus particularly, the presence of transependymal edema may be a predictor of unresolved hydrocephalus after resection. Obstructed hydrocephalus can be often resolved when the major CSF pathway become unobstructed after the removal of the mass effect.

Hemostatic agent is a widely used tool in neurosurgical daily practice. However, it has been reported that migration of Gelfoam fragments can cause occlusion of ETV or shunt, leading to hydrocephalus recurrence (6). Interestingly, we share several similar cases in our center. What these cases have in common is that the hemostatic agent migrate upward and cause occlusion of the CSF pathway in patients with a subtentorial tumor. Intraventricular hemostatic agent used in LVT surgery is not a independent predictor for postoperative CSF drainage in our series. The reason may be that the hemostatic agent has low density and is not easy to deposit and cause occlusion in the lateral ventricles. Despite the lack of evidence to support it, we advocate reducing the placement of hemostatic agent in the ventricles.

We found that subtotal resection was not a risk factor for postoperative CSF drainage. Clinically, complete tumor resection is our surgical goal. However, some tumors are difficult to completely resect because of their rich blood supply, nonclearly distinct boundaries, or tightly adhesive with normal brain tissue. To protect normal brain tissue and blood vessels, and to prevent severe postoperative intracranial edema and intracranial hypertension, our typical surgical goal is to achieve decompression and restore the CSF circulation pathway (23). Although residual tumor tissue may cause recurrence, slow-growing tumors do not generally cause acute intracranial hypertension. In addition, unlike other tumors, the choroid plexus tumor has the unique etiology of hydrocephalus due to the excess production of CSF. Normally, total resection without the complication of postoperative hemorrhage can solve the hydrocephalus. This study enrolled five cases of choroid plexus tumors, of which one case with the pathological result of papillary carcinoma receiving subtotal resection received postoperative EVD and VP shunt placement due to hydrocephalus. For patients with subtotal resection, a regular follow-up is required. A secondary resection can be considered for patients with tumor recurrence.

Numerous research studies have suggested that pediatric patients with subtentorial ventricle tumors can more easily develop postresection hydrocephalus than adult patients due to the characteristic of unique tumor pathology in each ages (24, 25). By contrast, for patients with LVTs, although a rather part of pediatric patients has primary symptomatic hydrocephalus, the common types of tumor in pediatric patients are benign, and most pediatric patients will not develop acute or persistent hydrocephalus after resection (26).

Perilesional edema was the only independent risk factor for VP shunt placement after surgery in our series after two cases of isolated hydrocephalus were excluded in the regression analysis. We believe that this may be related to the histology of the tumors. Perilesional edema is more common in malignant intracranial tumors than in benign ones. Intracranial malignant tumors are more likely to cause obstruction due to recurrence and impair CSF absorption due to the leptomeningeal metastases at the subarachnoid level and the high CSF protein content produced by disseminated tumor cells (27–29). Seven of the nine cases of the postoperative VP shunt were malignant. What is more, a rather part of patients with malignant tumors needs follow-up radiant therapy. It was reported that radiation-induced brain atrophy can exhibit mildly elevated CSF pressure because of impaired CSF flow and reduced reabsorption caused by fibrosis of the arachnoid granulations (30).

In the present study, two patients with LVTs located at the occipital angle of the lateral ventricle developed isolated hydrocephalus after resection: one patient had a glioblastoma that was difficult to completely resect. Recurrence of the residual tumor tissue causes adhesion and ventricle isolation. While the other patient with meningioma who received complete resection showed local brain tissue adherence and ventricle isolation. Ma et al. reported that excessive CSF loss by ventricular drainage can cause intracranial hemorrhage and ventricular wall adhesion, increasing the risk of isolated hydrocephalus (23). However, the patient with meningioma in our study did not receive a prophylactic EVD. Based on preoperative and postoperative MRI findings, we considered that this was related to the ventricle morphology around the tumor. The tumor with a large volume expanded the surrounding ventricle wall and brain tissues, while the other space of the ventricle was not enlarged and the space of the ventricle next to the tumor was relatively narrow. After tumor removal, the surrounding brain tissue collapsed and the wide basement of the tumor resulted in a large area of peritumoral brain tissue edema, which aggravated the partly ventricle stenosis, at last leading to the ventricle wall adhesion and developing into isolated hydrocephalus. For such brain tissue-wrapped tumors, we suggest timely postoperative imaging examination and adequate dehydration treatment for cerebral edema recedes. We also recommend that the distal end of the shunt tube be placed across the ventricle stenosis and the volume of CSF should be monitored in the case of ventricle collapse due to excessive drainage.

## Limitations

There are some limitations to our study. First, because our postoperative follow-up time varied from 3 months to 6 years, it remains unclear whether patients with a short follow-up time would develop hydrocephalus. This may have caused bias in our results. Second, because ETV was only performed on a few patients with LVTs in our center, the evaluation of the utility of ETV was limited. Finally, there are differences in surgical procedures and perioperative management between different medical centers, which may influence our statistical findings. Further prospective studies with larger samples are required to confirm our findings.

## Conclusion

Anterior invasion, transependymal edema, and large volume of postoperative IVH play a critical role in the development of postresection acute hydrocephalus in patients with LVTs. Prophylactic EVD and proper management of intracranial pressure are recommended for tumors with transependymal edema and invading the anterior part of the lateral ventricles. Patients with LVTs that showed perilesional edema on MRI were more likely to develop persistent hydrocephalus and need a VP shunt placement. Large triangular LVTs surrounded by a ventricle wall with a locally expanded ventricle are more likely to develop isolated hydrocephalus after surgery. These findings may help in identifying patients at risk of developing hydrocephalus after LVT surgery and the preoperative communication.

## Data Availability

The original contributions presented in the study are included in the article/Supplementary Material; further inquiries can be directed to the corresponding author/s.
